# Socioeconomic Disparities in All-Cause and Cause-Specific Mortality Rates among Municipalities in Japan, 1999–2019

**DOI:** 10.3390/ijerph17249213

**Published:** 2020-12-09

**Authors:** Tasuku Okui

**Affiliations:** Medical Information Center, Kyushu University Hospital, Fukuoka City 812-8582, Japan; okui.tasuku.509@m.kyushu-u.ac.jp; Tel.: +81-092-642-5882

**Keywords:** mortality rate, Japan, municipalities, health status disparities

## Abstract

Differences in all-cause and cause-specific mortality rates depending on municipal socioeconomic status (SES) in Japan have not been revealed over the last 20 years. This study exposes the difference in 1999 and 2019 using the Vital Statistics. All of the municipalities were grouped into five quintiles based on their SES, and standardized mortality ratio (SMR) of each municipal quintile compared with all of Japan was calculated for all-cause mortality and representative cause of deaths. As a result, although SMR for all-cause mortality for women tended to be lower in low SES quintiles in 1999, the reverse phenomenon was observed in 2019. Additionally, although SMR for all-cause of mortality for men was the lowest in the highest SES quintiles already in 1999, the difference in the SMR for all-cause mortality rates between the lowest and highest SES quintiles increased in 2019. The improvement of the SMR in the highest SES quintile and the deterioration in the lowest was also observed in representative types of cancer, heart disease, stroke, pneumonia, liver disease, and renal failure for men and women. Therefore, this study indicates a disparity in mortality depending on municipal SES enlarged in the last 20 years.

## 1. Introduction

Japan is one of the countries with the longest longevity worldwide, and its life expectancy is continuing to increase over time. Particularly, age-standardized mortality rates of a major cause of mortality such as cancer and cardiovascular diseases have decreased in recent decades [1]. On the other hand, there are regional disparities in mortality rate. According to a recent report investigating the difference in the change of life expectancy among prefectures in Japan [2], differences in life expectancy among prefectures has been growing in recent years. When we discuss regional mortality rate differences, variance in regional mortality rate depending on its SES is often of interest for public health practice. Many studies globally investigating the relationships between regional SES and all-cause or cause-specific mortality rate [3,4,5], as well as a disparity among SES, have been shown in several countries. Some studies also indicate association between Japan’s individual SES and mortality rates or health in recent years [6,7], and it is meaningful to examine whether there are differences in mortality rate depending on regional SES using recent Japan Vital Statistics data.

Numerous studies have investigated regional SES and mortality rate in Japan. One such study examined the relationship between municipal SES and all-cause and cause-specific mortality rate in Japan using the data of 1973–1977 and 1993–1998 [8]. This previous study revealed decreasing of relative importance of socioeconomic inequality in regional mortality of stroke, while that of injury and suicide increased during the periods examined. Furthermore, municipal deprivation level—which is determined by factors such as employment and household status of each municipality—and mortality rate for some types of cancer was shown to be associated in Japan using the Vital Statistics data from 2003 to 2007 [9]. Moreover, an epidemiological study using cohort data was conducted to determine the relationship between Japan’s municipal SES and mortality rate over the last few decades [6,10,11]. However, no study has examined the relationship between municipal SES and all-cause and cause-specific mortality data using Japan’s recent Vital Statistics, and the change in relationship during the last few decades is unknown. Although age-standardized morality rates of many disease types have decreased in recent decades [1], it is unknown whether the relationship between municipal SES and mortality rate changed for each cause of death. Therefore, this study investigated the mortality rate depending on municipal SES level using recent Vital Statistics in Japan and revealed the relationship change seen in recent decades. In addition, a previous study showed that the relationship between areal SES and mortality varied depending on sex for some cancer types [8]. In addition, trends in the prevalence of certain health behaviors differs depending on sex in the analyzed periods in Japan [12], and it is possible that the trends in the relationship between SES and mortality would also vary depending on sex for some diseases. Therefore, this study also focused on sex differences in the relationship between SES and mortality.

## 2. Materials and Methods

The Vital Statistics data in Japan in 1999 and 2019 were analyzed [13]. In addition, mortality data of major diseases by sex and year were obtained for each municipality, specifically all-cause mortality data; cancer in all sites; cancer of the stomach, colon, liver, pancreas and lung; heart disease; ischemic heart disease; stroke; pneumonia; liver disease; renal failure; natural death; injury; and suicide. The corresponding International Classification of Diseases (10th Revision) codes for each cause of death are as follows: cancer in all sites, C00–C97, stomach, C16; colorectal, C18‒C20; liver, C22; pancreas, C25; lung, C33–C34; heart disease, I01–I02.0, I05–I09, I20–I25, I27, and I30–I51; ischemic heart disease, I20–I25; stroke, I60–I69; pneumonia, J12–J18; liver disease, K70–K76; renal failure, N17–N19; natural death, R54; injury, V01–X59; and suicide, X60–X84 [14]. Each municipality’s population for each age group, sex, and year were extracted from data reported in the survey of population, demographics, and household number based on the basic resident register [15]. The data of age groups of 5-year increments from ages 0 to 79 and 80 or higher were included in population data.

Data on all municipalities in Japan were used for the analysis, and the total number of municipalities in Japan was 3256 in 1999 and 1742 in 2019. Each government ordinance-designated municipality was treated as one municipality. However, there were some municipalities whose SES information was missing, and those municipalities were excluded from the analysis.

Taxable income was used to determine SES of each municipality, as utilized in a previous study [8]. Although the previous study utilized educational level, as well [8], it was not used in this study; the latest educational level data for each municipality in recent years were not available, because the Census in Japan investigates educational level of each municipality every 10 years. Therefore, taxable income per capita in 2018 was used for the analysis [16]. Quintiles of taxable income per capita were calculated across all municipalities for each year, and each municipality was classified into quintile groups based on its taxable income level per capita. Then, mortality data for each cause of death and the population data were aggregated for each quintile.

To confirm the change in age-standardized mortality rate in the analyzed periods for each cause of death, age-standardized mortality rates for all of Japan were calculated in 1999 and 2019. The total population in 1999 was used as standard population, and direct method was used to calculate age-standardized mortality rates. Then, SMR for each quintile and its 95% confidence interval compared with Japan as a whole were calculated for each sex, year, and cause of death. All statistical analyses were conducted using R3.6.3 (R Core Team, Vienna, Austria: https://www.R-project.org/). 

## 3. Results

Table 1 indicates basic characteristics of each municipal SES quintile. Taxable income per capita evidently increased from quintile 1 to 5, as did mean population.

Table 2 shows Japan’s number of deaths and age-standardized mortality rates per 100,000 people for all-cause and each cause of mortality in 1999 and 2019. All the causes of mortality except pancreatic cancer and natural death decreased from 1999 to 2019 for both sexes.

Table 3 shows SMR for all-cause and each cause of death by municipal SES quintile in 1999 and 2019 for men. Disparity in the all-cause mortality rates between the lowest and highest SES increased from 1999 to 2019. Although SMR for cancer in all sites expanded with an increase in the municipal socioeconomic status in 1999, the SMR became the lowest within the highest SES quintile in 2019. Similar reversal phenomenon was also observed for stomach, colorectal, liver, and lung cancer. The reversal phenomenon was also observed for heart disease, whereas the trend for ischemic heart disease was opposite from heart disease overall. An increase in the degree of the disparity between the highest and lowest SES quintiles was also observed for pneumonia, liver disease, renal failure, injury, and suicide. A clear gradient in the SMR depending on areal SES was observed only for suicide in 1999, but it was observed for all-cause, stroke, renal failure, injury, and suicide in 2019.

Table 4 shows SMR for all-cause and each cause of death for women by municipal SES quintile in 1999 and 2019. Although SMR for all-cause mortality rate increased in the higher SES quintile in 1999, the SMR dropped to the lowest within the highest SES quintile in 2019. Additionally, a decrease of SMR in the highest SES quintile and an increase of SMR in the lowest SES quintile were observed for cancer in all sites, as well as cancer of the stomach, colon, liver, and pancreas. Trends of cardiovascular diseases were similar among men. Furthermore, reversal phenomenon for the relationship of SES and SMR was also observed for pneumonia, liver cancer, and renal failure. However, the disparity in mortality rate depending on municipal SES diminished for suicide. A clear gradient in the SMR depending on areal SES was not observed in 1999, but it was observed for renal failure in 2019. On the other hand, a trend toward an opposite gradient (the higher the SES, the higher the SMR) was observed for some cancer types in 1999, whereas the trend was not evident in 2019 among men or women.

## 4. Discussion

This study compared SMR among municipalities by their SES. The disparity depending on municipal SES was observed in 1999 for women only, and it is consistent with a previous study investigating the disparity from 1993 to 1998 [8]. However, in 2019, there was a disparity in the all-cause mortality rates for both sexes. The possible reason for the change in the SMR from 1999 to 2018 for each cause of death was discussed.

Regarding cancer mortality, although SMR for the highest SES quintile was greater than that for the lowest SES quintile in 1999 for men, the relationship reverted in 2019. Stomach, colorectal, and liver cancer were particularly lower in the municipalities with the lowest SES quantile for men and women in 1999. It is known that the colorectal cancer incidence rate rose from the 1970s in Japan [17], and a change in dietary patterns is said to have been a major factor. Therefore, it is considered that westernization of diets was more prevalent in municipalities with higher SES in 1999. Regarding liver cancer, alcohol use is known to correlate with the liver cancer trend in Japan [18,19]. Urbanization is known to be associated with increase of alcohol use for Japanese women [20], and excessive alcohol consumption was considered prevalent in urban areas [21]. Therefore, it is possible that alcohol use was related to higher liver cancer mortality in municipalities with high SES in 1999. However, age-standardized mortality rates for all noted cancer types were shown to decrease in the analyzed periods [1], and it is considered that degree of decrease of the mortality rate was greater in municipalities with higher SES. Although the reason for the larger decrease in mortality rates in municipalities with higher SES is considered to vary depending on cancer types, multiple factors such as change in dietary, drinking, and smoking prevalence are considered related. As one possible factor, participation rate of representative cancer screening was shown to be associated with individual SES in the data of 2001 [22,23]. Therefore, it is possible that a disparity in the participation rate depending on SES affected the mortality rate over the years. However, cancer screening for pancreatic and liver cancer is generally not conducted in Japan, and other factors are also considered to be related to the change in the SMRs. Smoking prevalence is a factor that is associated with mortality due to many cancer types [24]. Smoking prevalence is strongly associated with SES in Japan, and SES differences in smoking prevalence increased during the analyzed periods [25].

There was also a disparity in heart disease and stroke mortality among municipalities depending on their SES in 2019, while SMR was higher in higher SES quintiles for ischemic heart disease. The relationship between SES and stroke mortality is well known worldwide [26], and it is said that people with low SES tend to have risk factors for stroke (e.g., hypertension, hyperlipidemia, smoking, and obesity) and receive less medical care, which is considered to lead to the incidence of stroke. Heart disease and stroke are cardiovascular diseases, and similar factors are considered related to the mortality trends. As a factor, hypertension is an established risk factor for cardiovascular diseases [27]. Although multiple factors were noted as causes for the decrease in the stroke mortality rate in the late 20th century, change in blood pressure for Japanese people is said to be a major factor [28]. Systolic blood pressure has continuously decreased since the 1960s in Japan [29], while the treatment rate of hypertension has continued to increase over the analyzed period in Japan [29]. Therefore, the degree of the decrease in hypertension prevalence or the increase in the control rate of hypertension is considered to differ depending on municipal SES. Actually, hypertension prevalence and control rate is known to correlate with individual SES in Japan [30]. In addition, the trend of smoking prevalence is also considered related to the difference among municipalities [28], and decreasing rate of smoking prevalence over the years is shown to be associated with individual SES in Japan [25]. On the other hand, the trends in SMRs of ischemic heart disease were different from the other cardiovascular diseases. In a previous study using the data of the end of 20th century, the SMR of ischemic heart disease mortality was shown to be higher in the higher SES areas [8]. According to epidemiological studies, incidence of ischemic heart disease is known to have increased particularly in urban areas in the late 20th century in Japan [31,32]. As with some types of cancer, change in dietary habits is considered associated with the increase of the ischemic heart disease mortality [28], and it may have led to the higher mortality rates in municipalities with high SES. Although fat intake increased in the late 20th century in Japan, ischemic heart disease mortality did not increase very much [28]. To explain this phenomenon, it has been suggested that the adverse effects of dietary habits were overcome by a decrease in blood pressure levels and smoking prevalence [28]. Therefore, increased fat intake or the westernization of dietary habits is considered to be a cause of the difference in the trends between ischemic heart disease and the other types of cardiovascular diseases. However, the reasons for the increase in the disparity among quintiles from 1999 to 2019 both among men and women are unclear, and additional studies are warranted to explore this issue. Fat intake and ischemic heart disease mortality vary depending on birth cohorts in Japan [33,34]; thus, an analysis of differences in birth cohorts and their effects on ischemic heart disease mortality depending on areal SES would be helpful.

The increase in the disparity of SMR between the highest and the lowest SES quintiles from 1999 to 2019 was also observed for pneumonia, liver disease, renal failure, and injury for both sexes. No previous studies have investigated the difference in mortality rate for these causes depending on municipal SES in Japan, except for the previous study by Fukuda et al. [8]. However, several studies show the association between regional SES and mortality rate from these causes in other countries. In China, for instance, urban-rural injury mortality disparity appeared to decrease from 2010 to 2016 [35], and an increase of emergency institutions and personnel in rural areas is highlighted as a factor. It is known that there are also regional differences in the number of emergency institutions and personnel in Japan [36], and the disparity among municipalities depending on their SES might have increased during those periods. Regarding liver disease, there are similarities in the causes of liver cancer, with alcohol use and hepatitis virus prevalence considered related [19].

Regarding suicide, the mortality rate in Japan began decreasing from approximately 2010 [1]. In addition, Japan’s municipal unemployment and mortality rates were shown to be connected [37]. However, the present study found that regional disparity in suicide increased for men but not for women, and there is a possibility that decreasing suicide mortality rate has contributed to decreased disparity for women. According to a previous study investigating socioeconomic determinants for suicide using national statistics data of 1957–2009 [38], the association of suicide with sociological factors (divorce and fertility rates) was stronger for women than those with economic factors. Therefore, the association between SES and suicide mortality rate is considered to vary by sex in Japan.

This study revealed that the degree of disparity increased from 1999 to 2019 in all-cause mortality. Although the previous study by Fukuda et al. showed socioeconomic disparity in various kinds of cause of mortality among municipalities decreased form the 1970s to the 1990s in Japan [8], it was shown that the degree of disparity increased again in the last 20 years. The values of SMR in the highest SES quintile ameliorated for multiple types of cause of mortality in the periods, and multiple factors are considered related to this phenomenon. It is well known SES is related to various kinds of health-related behaviors, such as smoking, participation in cancer screening, and hypertension control in the current Japanese society [21,29,39,40,41]. Health literacy is a factor associated with these behaviors and with SES. Individuals with low SES tend to have low health literacy [42], and low health literacy is considered to be related to unhealthy behaviors. Therefore, not only public awareness for healthy lifestyle behaviors, but also a method for improving health literacy in individuals with low SES is needed to improve the current trends in Japan. To increase the health literacy of individuals in low SES regions, an increase in the opportunities for education on healthy behaviors starting in childhood is one method. Furthermore, allocating more medical resources to individuals with low SES is another method, and lowering medical fees or making participation of cancer screening free of charge for those people is one example. In addition, a policy aiming at reducing regional differences in medical resources, such as number of hospitals or physicians, may also needed. On the other hand, unhealthy lifestyle behaviors are not necessarily related to low SES in Japan. According to a 2001 national survey, unhealthy lifestyle behaviors are more common in urban areas, particularly among women [21], and habitual alcohol drinking is more common in individuals of high SES in Japan [40]. It is considered that these factors were related to the mortality of some diseases even in 2019, and municipalities in the lowest SES quintile had the lowest SMR for some cancer types among women. Actually, lifestyle behaviors including dietary habits were considered factors related to a higher mortality rate for breast and colorectal cancer in urban areas at the end of the 20th century [43]. The prevalence of unhealthy lifestyle behavior changes along birth cohorts in Japan [12], and the relationship between SES and disease mortality or unhealthy behaviors also varies depending on birth cohorts. Therefore, a reduction in unhealthy lifestyle behaviors might be more urgently needed for individuals in high SES areas, particularly among older age groups. In order to identify the accurate reason for the current trend of the disparity in Japan, differences in health-related behavior or socioeconomic environments need to be investigated among the SES quintiles. In addition, there is a possibility that regional SES is becoming larger over the birth cohorts. Actually, a possibility was pointed out that younger ages might face more socioeconomic disparities compared with middle or older ages from the previous study by Fukuda et al. [11]. A study investigating mortality difference by regional SES over birth cohorts will be meaningful in the future.

As a limitation of this study, only obtain total mortality data throughout all ages for each municipality were obtained, and the mortality data for each age group were not obtained. Therefore, only SMR was calculated for investigating the degree of disparity among municipalities. The degree of disparity might differ depending on ages or birth cohorts. As another limitation, the total number of municipalities changed between 1999 to 2019. Many municipalities with small populations were integrated to become larger municipalities in the 2000s. Although it is difficult to evaluate the effect of this integration, there is a possibility that it contributed to a reduction in the differences in the SMRs among the quintiles for some causes of mortality. Moreover, the SES of an area generally contains multiple aspects of an area, such as social deprivation level or financial capabilities. This study focused only on taxable income per capita as an indicator of SES, so an analysis using other types of SES information in the future would be useful. Furthermore, only area level data were analyzed in this study, and an epidemiological study focusing on mortality differences by individual SES would also be helpful. If the taxable income varies within each municipality, the results derived from the data of areal SES might differ from that for individuals’ SES. However, the data of the Vital Statistics in Japan were analyzed, and the result of this study represents the country’s overall trend.

## 5. Conclusions

The difference in SMR for all-cause and cause-specific mortality depending on municipal SES in 1999 and 2019 was revealed. As a result, although SMR for all-cause mortality tended to be rather lower in low SES quintiles in 1999 for women, the reverse phenomenon was observed in 2019. Moreover, although SMR for all-cause of mortality was already the lowest in the highest SES quintiles for men in 1999, the difference in the SMR for all-cause mortality rates between the lowest and highest SES quintiles increased in 2019. The improvement of the SMR in the highest SES quintile and the deterioration in the lowest SES quintile was also observed in representative types of cancer, heart disease, stroke, pneumonia, liver disease, and renal failure for both sexes. Therefore, this study indicates a disparity in mortality depending on municipal SES enlarged in the last 20 years.

## Figures and Tables

**Table 1:** Basic characteristics of each municipal socioeconomic status quintile.

		Characteristics			
Year	Municipal Socioeconomic Status	Number of Municipalities	Taxable Income Per Capita (Thousand Yen) *	Male Population * (Thousand Persons)	Female Population * (Thousand Persons)
1999					
	Quintile 1 (lowest)	650	825.6 (758.7–877.4)	2.80 (1.79–4.65)	3.07 (1.95–5.06)
	Quintile 2	650	1003.7 (969.7–1042.4)	3.55 (2.18–5.77)	3.84 (2.38–6.32)
	Quintile 3	651	1151.7 (1114.8–1186.4)	4.69 (2.81–8.76)	4.97 (3.04–9.26)
	Quintile 4	649	1321.6 (1274.7–1365.9)	7.77 (4.14–17.60)	8.04 (4.35–18.39)
	Quintile 5 (highest)	651	1585.5 (1490.1–1727.6)	22.99 (9.72–59.38)	23.15 (10.07–59.82)
2019					
	Quintile 1 (lowest)	348	887.5 (823.6–939.4)	3.71 (1.70–7.62)	4.04 (1.86–8.37)
	Quintile 2	348	1057.8 (1025.2–1093.4)	7.70 (3.36–16.14)	8.13 (3.58–17.54)
	Quintile 3	348	1196.9 (1164.4–1235.4)	12.74 (4.73–24.58)	13.59 (5.10–25.83)
	Quintile 4	348	1343.8 (1305.6–1390.5)	21.29 (8.51–45.24)	21.64 (8.88–47.00)
	Quintile 5 (highest)	348	1599.0 (1507.9–1774.9)	45.40 (19.85–108.71)	44.96 (20.55–111.44)

* Median (Interquartile range).

**Table 2:** Japan’s number of deaths and age-standardized mortality rates per 100,000 people for all-cause and each cause of mortality in 1999 and 2019.

		1999		2019	
Sex	Cause of Mortality	Number of Deaths	Age-Standardized Mortality Rate Per 100,000 Persons	Number of Deaths	Age-Standardized Mortality Rate Per 100,000 Persons
Men					
	All-cause	534,076	1062.4	707,062	756.7
	Cancer in all sites	175,802	336.4	220,329	240.5
	Stomach cancer	32,784	63.0	28,042	30.4
	Colorectal cancer	19,415	37.0	27,415	30.8
	Liver cancer	23,490	42.5	16,749	18.3
	Pancreatic cancer	10,203	19.3	18,124	20.5
	Lung cancer	37,932	74.0	53,336	57.6
	Heart disease	73,932	150.0	98,185	104.6
	Ischemic heart disease	39,468	79.1	39,571	44.1
	Stroke	66,423	137.9	51,766	55.2
	Pneumonia	49,889	110.4	53,064	51.7
	Liver disease	11,445	20.1	11,233	14.4
	Renal failure	8304	17.8	13,572	13.5
	Natural death	6600	15.9	31,720	29.3
	Injury	25,455	47.1	22,375	25.4
	Suicide	22,124	36.8	13,637	21.0
Women					
	All-cause	447,160	579.9	673,547	446.2
	Cancer in all sites	114,736	157.3	156,082	128.6
	Stomach cancer	17,887	24.2	14,888	11.4
	Colorectal cancer	15,945	21.6	24,004	18.8
	Liver cancer	10,324	14.1	8514	5.9
	Pancreatic cancer	8450	11.4	18,232	14.5
	Lung cancer	14,241	19.2	22,055	16.9
	Heart disease	77,096	97.0	109,489	65.4
	Ischemic heart disease	34,426	43.6	27,733	18.1
	Stroke	72,533	91.3	54,783	34.7
	Pneumonia	44,090	54.0	42,441	23.9
	Liver disease	5129	7.0	6037	5.2
	Renal failure	9394	11.7	13,071	7.7
	Natural death	16,229	19.2	90,138	46.5
	Injury	14,519	19.6	16,779	11.8
	Suicide	8983	13.4	5752	8.3

**Table 3:** SMR for all-cause and each cause of death by municipal SES quintile in 1999 and 2019 for men.

		Municipal Socioeconomic Status				
		Quintile 1 (Lowest)	Quintile 2	Quintile 3	Quintile 4	Quintile 5 (Highest)
Cause of Death	Year	SMR (95% CI) *	SMR (95% CI) *	SMR (95% CI) *	SMR (95% CI) *	SMR (95% CI) *
All-cause						
	1999	1.02 (1.02, 1.03)	1.01 (1.01, 1.02)	1.01 (1.00, 1.02)	1.01 (1.01, 1.01)	0.99 (0.98, 0.99)
	2019	1.06 (1.06, 1.07)	1.05 (1.04, 1.05)	1.04 (1.03, 1.04)	1.01 (1.01, 1.02)	0.97 (0.97, 0.97)
Cancer in all sites						
	1999	0.93 (0.91, 0.94)	0.95 (0.94, 0.97)	0.98 (0.97, 0.99)	1.00 (1.00, 1.01)	1.01 (1.01, 1.02)
	2019	1.00 (0.98, 1.01)	1.01 (1.00, 1.02)	1.04 (1.03, 1.04)	1.02 (1.01, 1.02)	0.98 (0.98, 0.98)
Stomach cancer						
	1999	0.84 (0.81, 0.87)	0.92 (0.89, 0.94)	1.01 (0.99, 1.03)	1.01 (1.00, 1.03)	1.02 (1.01, 1.03)
	2019	0.94 (0.90, 0.98)	1.02 (0.99, 1.05)	1.05 (1.03, 1.07)	1.04 (1.03, 1.06)	0.97 (0.96, 0.98)
Colorectal cancer						
	1999	0.83 (0.79, 0.86)	0.90 (0.87, 0.93)	0.92 (0.89, 0.95)	1.00 (0.98, 1.02)	1.04 (1.03, 1.06)
	2019	1.00 (0.97, 1.04)	0.99 (0.96, 1.01)	1.05 (1.03, 1.08)	1.00 (0.98, 1.01)	0.99 (0.98, 1.00)
Liver cancer						
	1999	0.79 (0.76, 0.83)	0.88 (0.86, 0.91)	0.95 (0.92, 0.97)	1.01 (0.99, 1.03)	1.04 (1.02, 1.05)
	2019	0.97 (0.92, 1.01)	1.02 (0.99, 1.06)	1.05 (1.02, 1.08)	1.06 (1.04, 1.08)	0.96 (0.94, 0.97)
Pancreatic cancer						
	1999	1.00 (0.94, 1.05)	1.05 (1.00, 1.10)	1.01 (0.97, 1.05)	1.02 (1.00, 1.05)	0.98 (0.96, 1.00)
	2019	0.98 (0.93, 1.02)	1.00 (0.97, 1.04)	1.00 (0.97, 1.03)	1.03 (1.01, 1.05)	0.99 (0.97, 1.00)
Lung cancer						
	1999	0.96 (0.94, 0.99)	0.99 (0.97, 1.02)	1.00 (0.97, 1.02)	1.00 (0.99, 1.01)	1.00 (0.99, 1.01)
	2019	1.00 (0.97, 1.02)	1.02 (1.00, 1.04)	1.06 (1.04, 1.07)	1.03 (1.01, 1.04)	0.97 (0.96, 0.98)
Heart disease						
	1999	0.99 (0.97, 1.01)	1.00 (0.98, 1.01)	0.99 (0.98, 1.01)	1.01 (1.00, 1.02)	1.00 (0.99, 1.00)
	2019	1.06 (1.04, 1.08)	1.06 (1.04, 1.07)	1.06 (1.05, 1.07)	1.00 (0.99, 1.00)	0.97 (0.96, 0.98)
Ischemic heart disease						
	1999	0.91 (0.88, 0.94)	0.91 (0.89, 0.93)	0.91 (0.89, 0.93)	0.96 (0.95, 0.98)	1.05 (1.04, 1.06)
	2019	0.82 (0.79, 0.85)	0.84 (0.82, 0.86)	0.94 (0.92, 0.96)	1.00 (0.98, 1.01)	1.06 (1.05, 1.07)
Stroke						
	1999	1.05 (1.03, 1.07)	1.05 (1.03, 1.07)	1.07 (1.05, 1.08)	1.01 (1.00, 1.02)	0.97 (0.96, 0.97)
	2019	1.16 (1.13, 1.19)	1.13 (1.11, 1.15)	1.08 (1.06, 1.09)	1.02 (1.01, 1.03)	0.93 (0.92, 0.94)
Pneumonia						
	1999	1.03 (1.01, 1.05)	1.01 (0.99, 1.03)	0.97 (0.96, 0.99)	1.01 (0.99, 1.02)	0.99 (0.99, 1.00)
	2019	1.10 (1.08, 1.13)	1.04 (1.02, 1.06)	1.02 (1.01, 1.04)	1.03 (1.02, 1.04)	0.96 (0.95, 0.97)
Liver disease						
	1999	1.00 (0.94, 1.05)	0.93 (0.88, 0.97)	0.87 (0.84, 0.91)	0.98 (0.95, 1.00)	1.03 (1.01, 1.05)
	2019	1.18 (1.11, 1.25)	1.04 (1.00, 1.09)	0.93 (0.89, 0.96)	0.99 (0.96, 1.01)	1.00 (0.98, 1.02)
Renal failure						
	1999	1.00 (0.94, 1.06)	0.97 (0.92, 1.02)	1.03 (0.99, 1.07)	1.01 (0.97, 1.04)	0.99 (0.97, 1.01)
	2019	1.15 (1.10, 1.21)	1.07 (1.03, 1.11)	1.07 (1.04, 1.10)	1.06 (1.04, 1.09)	0.92 (0.91, 0.94)
Natural death						
	1999	1.15 (1.08, 1.21)	1.07 (1.02, 1.13)	1.21 (1.15, 1.26)	1.07 (1.03, 1.10)	0.90 (0.88, 0.92)
	2019	1.06 (1.03, 1.09)	1.00 (0.98, 1.03)	1.01 (0.99, 1.03)	0.95 (0.94, 0.97)	1.01 (1.00, 1.02)
Injury						
	1999	1.27 (1.23, 1.32)	1.29 (1.25, 1.32)	1.22 (1.19, 1.25)	1.08 (1.06, 1.10)	0.87 (0.86, 0.88)
	2019	1.32 (1.27, 1.36)	1.25 (1.22, 1.29)	1.09 (1.06, 1.11)	1.04 (1.02, 1.05)	0.89 (0.88, 0.90)
Suicide						
	1999	1.38 (1.33, 1.43)	1.21 (1.17, 1.25)	1.07 (1.04, 1.10)	1.01 (0.99, 1.03)	0.93 (0.92, 0.94)
	2019	1.42 (1.35, 1.49)	1.23 (1.18, 1.28)	1.09 (1.06, 1.13)	1.04 (1.02, 1.06)	0.91 (0.89, 0.92)

* SMR, standardized mortality ratio; CI, confidence interval.

**Table 4:** SMR for all-cause and each cause of death by municipal SES quintile in 1999 and 2019 for women.

		Municipal Socioeconomic Status				
		Quintile 1 (Lowest)	Quintile 2	Quintile 3	Quintile 4	Quintile 5 (highest)
Cause of Death	Year	SMR (95% CI) *	SMR (95% CI) *	SMR (95% CI) *	SMR (95% CI) *	SMR (95% CI) *
All-cause						
	1999	0.97 (0.96, 0.98)	0.99 (0.98, 0.99)	0.99 (0.98, 0.99)	1.01 (1.00, 1.01)	1.01 (1.00, 1.01)
	2019	1.02 (1.02, 1.03)	1.04 (1.03, 1.04)	1.04 (1.03, 1.04)	1.01 (1.01, 1.02)	0.97 (0.97, 0.97)
Cancer in all sites						
	1999	0.89 (0.88, 0.91)	0.92 (0.91, 0.93)	0.95 (0.93, 0.96)	0.99 (0.98, 1.00)	1.04 (1.03, 1.04)
	2019	0.93 (0.92, 0.95)	0.98 (0.97, 0.99)	1.02 (1.01, 1.02)	1.01 (1.01, 1.02)	1.00 (0.99, 1.00)
Stomach cancer						
	1999	0.79 (0.75, 0.82)	0.88 (0.84, 0.91)	0.98 (0.95, 1.01)	1.01 (0.99, 1.04)	1.04 (1.03, 1.05)
	2019	0.90 (0.86, 0.95)	1.04 (1.01, 1.08)	1.03 (1.00, 1.06)	1.02 (1.00, 1.04)	0.99 (0.97, 1.00)
Colorectal cancer						
	1999	0.81 (0.78, 0.85)	0.91 (0.87, 0.94)	0.95 (0.92, 0.98)	0.97 (0.95, 0.99)	1.06 (1.04, 1.07)
	2019	0.93 (0.90, 0.97)	0.95 (0.93, 0.98)	1.03 (1.00, 1.05)	1.02 (1.00, 1.03)	1.00 (0.99, 1.01)
Liver cancer						
	1999	0.87 (0.82, 0.91)	0.90 (0.86, 0.95)	0.91 (0.87, 0.95)	0.99 (0.96, 1.02)	1.05 (1.03, 1.07)
	2019	0.99 (0.93, 1.05)	0.96 (0.91, 1.00)	1.04 (1.00, 1.08)	1.05 (1.02, 1.08)	0.97 (0.95, 0.99)
Pancreatic cancer						
	1999	0.91 (0.86, 0.97)	1.01 (0.96, 1.06)	0.98 (0.94, 1.02)	1.00 (0.97, 1.03)	1.01 (0.99, 1.03)
	2019	0.94 (0.90, 0.98)	1.01 (0.98, 1.04)	1.06 (1.04, 1.09)	0.98 (0.96, 1.00)	1.00 (0.98, 1.01)
Lung cancer						
	1999	0.97 (0.93, 1.01)	0.89 (0.85, 0.93)	0.90 (0.87, 0.93)	0.96 (0.94, 0.99)	1.05 (1.04, 1.07)
	2019	0.90 (0.86, 0.94)	0.92 (0.90, 0.95)	0.96 (0.94, 0.99)	1.04 (1.02, 1.06)	1.01 (1.00, 1.03)
Heart disease						
	1999	0.98 (0.97, 1.00)	0.96 (0.95, 0.98)	0.98 (0.97, 1.00)	1.01 (1.00, 1.02)	1.01 (1.00, 1.01)
	2019	1.04 (1.03, 1.06)	1.06 (1.05, 1.07)	1.07 (1.06, 1.08)	1.02 (1.01, 1.03)	0.95 (0.95, 0.96)
Ischemic heart disease						
	1999	0.92 (0.89, 0.94)	0.92 (0.89, 0.94)	0.92 (0.90, 0.94)	0.98 (0.97, 1.00)	1.05 (1.04, 1.06)
	2019	0.85 (0.82, 0.89)	0.85 (0.83, 0.87)	0.97 (0.95, 0.99)	0.99 (0.98, 1.01)	1.06 (1.05, 1.07)
Stroke						
	1999	0.96 (0.95, 0.98)	1.05 (1.03, 1.06)	1.04 (1.03, 1.06)	1.04 (1.03, 1.05)	0.97 (0.96, 0.98)
	2019	1.13 (1.10, 1.15)	1.16 (1.14, 1.18)	1.08 (1.06, 1.09)	1.03 (1.01, 1.04)	0.92 (0.91, 0.92)
Pneumonia						
	1999	0.99 (0.97, 1.02)	0.96 (0.94, 0.98)	0.93 (0.91, 0.95)	0.99 (0.98, 1.01)	1.03 (1.02, 1.03)
	2019	1.09 (1.06, 1.12)	1.05 (1.03, 1.08)	1.07 (1.05, 1.08)	1.03 (1.01, 1.04)	0.95 (0.94, 0.95)
Liver disease						
	1999	0.93 (0.86, 1.01)	0.83 (0.77, 0.88)	0.95 (0.89, 1.00)	0.96 (0.92, 1.00)	1.06 (1.03, 1.08)
	2019	1.02 (0.95, 1.10)	1.04 (0.98, 1.10)	1.02 (0.98, 1.07)	1.03 (0.99, 1.06)	0.97 (0.95, 1.00)
Renal failure						
	1999	0.98 (0.92, 1.03)	0.95 (0.90, 0.99)	0.93 (0.90, 0.97)	1.04 (1.01, 1.07)	1.01 (0.99, 1.03)
	2019	1.20 (1.15, 1.25)	1.13 (1.09, 1.17)	1.10 (1.07, 1.13)	1.05 (1.03, 1.08)	0.89 (0.88, 0.91)
Natural death						
	1999	1.05 (1.01, 1.09)	1.18 (1.14, 1.21)	1.16 (1.13, 1.20)	1.06 (1.04, 1.08)	0.90 (0.89, 0.91)
	2019	1.03 (1.02, 1.05)	1.00 (0.99, 1.02)	1.02 (1.01, 1.03)	0.95 (0.94, 0.95)	1.02 (1.01, 1.02)
Injury						
	1999	0.99 (0.95, 1.03)	1.13 (1.09, 1.17)	1.09 (1.06, 1.13)	1.05 (1.03, 1.08)	0.94 (0.93, 0.96)
	2019	1.10 (1.06, 1.15)	1.08 (1.04, 1.11)	1.10 (1.07, 1.13)	1.02 (1.00, 1.04)	0.93 (0.92, 0.95)
Suicide						
	1999	1.20 (1.14, 1.27)	1.13 (1.07, 1.18)	1.06 (1.02, 1.11)	0.92 (0.89, 0.95)	0.98 (0.96, 1.00)
	2019	1.06 (0.96, 1.15)	1.00 (0.94, 1.06)	1.00 (0.95, 1.05)	0.99 (0.95, 1.02)	1.00 (0.98, 1.02)

* SMR, standardized mortality ratio; CI, confidence interval.
