# Erratum: Okui, T. Socioeconomic Disparities in All-Cause and Cause-Specific Mortality Rates among Municipalities in Japan, 1999–2019. *Int. J. Environ. Res. Public Health* 2020, *17*, 9213

**DOI:** 10.3390/ijerph18115781

**Published:** 2021-05-27

**Authors:** Tasuku Okui

**Affiliations:** Medical Information Center, Kyushu University Hospital, Fukuoka City 812-8582, Japan; okui.tasuku.509@m.kyushu-u.ac.jp; Tel.: +81-092-642-5882

The author wants to correct the values of the 95% confidence interval of the standardized mortality ratio (SMR) in Table 3 and Table 4 in the following paper [1].

## Figures and Tables

**Table 3:** SMR for all-cause and each cause of death by municipal SES quintile in 1999 and 2019 for men.

		Municipal Socioeconomic Status				
		Quintile 1 (Lowest)	Quintile 2	Quintile 3	Quintile 4	Quintile 5 (Highest)
Cause of Death	Year	SMR (95% CI) *	SMR (95% CI) *	SMR (95% CI) *	SMR (95% CI) *	SMR (95% CI) *
All cause						
	1999	1.02 (1.01, 1.03)	1.01 (1.00, 1.02)	1.01 (1.00, 1.02)	1.01 (1.00, 1.02)	0.99 (0.98, 0.99)
	2019	1.06 (1.05, 1.08)	1.05 (1.04, 1.05)	1.04 (1.03, 1.04)	1.01 (1.01, 1.02)	0.97 (0.97, 0.97)
Cancer in all sites						
	1999	0.93 (0.91, 0.95)	0.95 (0.94, 0.97)	0.98 (0.96, 0.99)	1.00 (0.99, 1.01)	1.01 (1.01, 1.02)
	2019	1.00 (0.98, 1.02)	1.01 (1.00, 1.02)	1.04 (1.02, 1.05)	1.02 (1.01, 1.03)	0.98 (0.97, 0.99)
Stomach cancer						
	1999	0.84 (0.80, 0.88)	0.92 (0.88, 0.96)	1.01 (0.97, 1.04)	1.01 (0.99, 1.04)	1.02 (1.00, 1.03)
	2019	0.94 (0.89, 0.99)	1.02 (0.98, 1.06)	1.05 (1.02, 1.09)	1.04 (1.02, 1.07)	0.97 (0.95, 0.98)
Colorectal cancer						
	1999	0.83 (0.77, 0.88)	0.90 (0.85, 0.95)	0.92 (0.88, 0.97)	1.00 (0.97, 1.03)	1.04 (1.02, 1.06)
	2019	1.00 (0.95, 1.06)	0.99 (0.95, 1.03)	1.05 (1.02, 1.09)	1.00 (0.97, 1.02)	0.99 (0.97, 1.00)
Liver cancer						
	1999	0.79 (0.74, 0.84)	0.88 (0.84, 0.93)	0.95 (0.91, 0.99)	1.01 (0.98, 1.04)	1.04 (1.02, 1.05)
	2019	0.97 (0.90, 1.04)	1.02 (0.97, 1.08)	1.05 (1.01, 1.09)	1.06 (1.03, 1.09)	0.96 (0.94, 0.98)
Pancreatic cancer						
	1999	1.00 (0.92, 1.09)	1.05 (0.98, 1.12)	1.01 (0.95, 1.08)	1.02 (0.98, 1.07)	0.98 (0.96, 1.01)
	2019	0.98 (0.91, 1.05)	1.00 (0.95, 1.06)	1.00 (0.96, 1.04)	1.03 (1.00, 1.06)	0.99 (0.97, 1.01)
Lung cancer						
	1999	0.96 (0.92, 1.00)	0.99 (0.96, 1.03)	1.00 (0.96, 1.03)	1.00 (0.98, 1.02)	1.00 (0.99, 1.02)
	2019	1.00 (0.96, 1.04)	1.02 (0.99, 1.05)	1.06 (1.03, 1.08)	1.03 (1.01, 1.04)	0.97 (0.96, 0.98)
Heart disease						
	1999	0.99 (0.96, 1.02)	1.00 (0.97, 1.02)	0.99 (0.97, 1.01)	1.01 (0.99, 1.02)	1.00 (0.99, 1.01)
	2019	1.06 (1.03, 1.09)	1.06 (1.03, 1.08)	1.06 (1.04, 1.08)	1.00 (0.98, 1.01)	0.97 (0.96, 0.98)
Ischemic heart disease						
	1999	0.91 (0.87, 0.95)	0.91 (0.88, 0.94)	0.91 (0.88, 0.94)	0.96 (0.94, 0.99)	1.05 (1.03, 1.06)
	2019	0.82 (0.78, 0.86)	0.84 (0.81, 0.87)	0.94 (0.91, 0.97)	1.00 (0.98, 1.02)	1.06 (1.05, 1.07)
Stroke						
	1999	1.05 (1.01, 1.08)	1.05 (1.02, 1.08)	1.07 (1.04, 1.09)	1.01 (1.00, 1.03)	0.97 (0.96, 0.98)
	2019	1.16 (1.11, 1.20)	1.13 (1.10, 1.16)	1.08 (1.05, 1.10)	1.02 (1.00, 1.04)	0.93 (0.92, 0.94)
Pneumonia						
	1999	1.03 (1.00, 1.07)	1.01 (0.98, 1.04)	0.97 (0.95, 1.00)	1.01 (0.99, 1.03)	0.99 (0.98, 1.01)
	2019	1.10 (1.06, 1.14)	1.04 (1.01, 1.07)	1.02 (1.00, 1.05)	1.03 (1.01, 1.05)	0.96 (0.95, 0.97)
Liver disease						
	1999	1.00 (0.92, 1.08)	0.93 (0.86, 1.00)	0.87 (0.82, 0.93)	0.98 (0.94, 1.02)	1.03 (1.00, 1.05)
	2019	1.18 (1.08, 1.28)	1.04 (0.98, 1.11)	0.93 (0.88, 0.98)	0.99 (0.95, 1.02)	1.00 (0.98, 1.03)
Renal failure						
	1999	1.00 (0.92, 1.09)	0.97 (0.90, 1.05)	1.03 (0.96, 1.10)	1.01 (0.96, 1.05)	0.99 (0.96, 1.02)
	2019	1.15 (1.07, 1.24)	1.07 (1.01, 1.13)	1.07 (1.02, 1.12)	1.06 (1.03, 1.10)	0.92 (0.90, 0.95)
Natural death						
	1999	1.15 (1.05, 1.25)	1.07 (0.99, 1.16)	1.21 (1.13, 1.29)	1.07 (1.01, 1.12)	0.90 (0.86, 0.93)
	2019	1.06 (1.01, 1.11)	1.00 (0.97, 1.04)	1.01 (0.98, 1.04)	0.95 (0.93, 0.98)	1.01 (1.00, 1.03)
Injury						
	1999	1.27 (1.21, 1.34)	1.29 (1.23, 1.34)	1.22 (1.18, 1.27)	1.08 (1.05, 1.11)	0.87 (0.85, 0.88)
	2019	1.32 (1.25, 1.39)	1.25 (1.20, 1.30)	1.09 (1.05, 1.13)	1.04 (1.01, 1.06)	0.89 (0.87, 0.91)
Suicide						
	1999	1.38 (1.31, 1.46)	1.21 (1.15, 1.27)	1.07 (1.02, 1.12)	1.01 (0.98, 1.04)	0.93 (0.91, 0.94)
	2019	1.42 (1.31, 1.53)	1.23 (1.16, 1.30)	1.09 (1.04, 1.14)	1.04 (1.00, 1.08)	0.91 (0.88, 0.93)

* SMR, standardized mortality ratio; CI, confidence interval.

**Table 4:** SMR for all-cause and each cause of death by municipal SES quintile in 1999 and 2019 for women.

		Municipal Socioeconomic Status				
		Quintile 1 (Lowest)	Quintile 2	Quintile 3	Quintile 4	Quintile 5 (Highest)
Cause of Death	Year	SMR (95% CI) *	SMR (95% CI) *	SMR (95% CI) *	SMR (95% CI) *	SMR (95% CI) *
All cause						
	1999	0.97 (0.96, 0.98)	0.99 (0.98, 1.00)	0.99 (0.98, 0.99)	1.01 (1.00, 1.01)	1.01 (1.00, 1.01)
	2019	1.02 (1.01, 1.04)	1.04 (1.03, 1.04)	1.04 (1.03, 1.04)	1.01 (1.01, 1.02)	0.97 (0.97, 0.98)
Cancer in all sites						
	1999	0.89 (0.87, 0.92)	0.92 (0.90, 0.94)	0.95 (0.93, 0.96)	0.99 (0.97, 1.00)	1.04 (1.03, 1.05)
	2019	0.93 (0.91, 0.95)	0.98 (0.97, 1.00)	1.02 (1.00, 1.03)	1.01 (1.00, 1.02)	1.00 (0.99, 1.01)
Stomach cancer						
	1999	0.79 (0.73, 0.84)	0.88 (0.83, 0.92)	0.98 (0.94, 1.03)	1.01 (0.98, 1.05)	1.04 (1.02, 1.06)
	2019	0.90 (0.83, 0.97)	1.04 (0.99, 1.10)	1.03 (0.99, 1.07)	1.02 (0.99, 1.05)	0.99 (0.96, 1.01)
Colorectal cancer						
	1999	0.81 (0.76, 0.87)	0.91 (0.86, 0.96)	0.95 (0.90, 1.00)	0.97 (0.94, 1.00)	1.06 (1.03, 1.08)
	2019	0.93 (0.88, 0.99)	0.95 (0.91, 1.00)	1.03 (0.99, 1.06)	1.02 (0.99, 1.04)	1.00 (0.98, 1.02)
Liver cancer						
	1999	0.87 (0.79, 0.94)	0.90 (0.84, 0.97)	0.91 (0.85, 0.97)	0.99 (0.95, 1.04)	1.05 (1.02, 1.08)
	2019	0.99 (0.90, 1.09)	0.96 (0.89, 1.03)	1.04 (0.98, 1.10)	1.05 (1.01, 1.10)	0.97 (0.94, 1.00)
Pancreatic cancer						
	1999	0.91 (0.83, 1.00)	1.01 (0.94, 1.09)	0.98 (0.92, 1.05)	1.00 (0.95, 1.05)	1.01 (0.98, 1.04)
	2019	0.94 (0.88, 1.00)	1.01 (0.96, 1.06)	1.06 (1.02, 1.10)	0.98 (0.95, 1.01)	1.00 (0.98, 1.02)
Lung cancer						
	1999	0.97 (0.91, 1.04)	0.89 (0.84, 0.95)	0.90 (0.85, 0.95)	0.96 (0.93, 1.00)	1.05 (1.03, 1.08)
	2019	0.90 (0.85, 0.96)	0.92 (0.88, 0.97)	0.96 (0.93, 1.00)	1.04 (1.01, 1.07)	1.01 (0.99, 1.03)
Heart disease						
	1999	0.98 (0.96, 1.01)	0.96 (0.94, 0.99)	0.98 (0.96, 1.00)	1.01 (1.00, 1.03)	1.01 (1.00, 1.02)
	2019	1.04 (1.02, 1.07)	1.06 (1.04, 1.08)	1.07 (1.06, 1.09)	1.02 (1.01, 1.03)	0.95 (0.94, 0.96)
Ischemic heart disease						
	1999	0.92 (0.88, 0.96)	0.92 (0.88, 0.95)	0.92 (0.89, 0.96)	0.98 (0.96, 1.00)	1.05 (1.03, 1.06)
	2019	0.85 (0.81, 0.90)	0.85 (0.81, 0.88)	0.97 (0.94, 1.01)	0.99 (0.97, 1.02)	1.06 (1.04, 1.08)
Stroke						
	1999	0.96 (0.94, 0.99)	1.05 (1.02, 1.07)	1.04 (1.02, 1.07)	1.04 (1.03, 1.06)	0.97 (0.96, 0.98)
	2019	1.13 (1.09, 1.17)	1.16 (1.13, 1.19)	1.08 (1.05, 1.10)	1.03 (1.01, 1.04)	0.92 (0.91, 0.93)
Pneumonia						
	1999	0.99 (0.96, 1.03)	0.96 (0.93, 0.99)	0.93 (0.90, 0.96)	0.99 (0.97, 1.01)	1.03 (1.01, 1.04)
	2019	1.09 (1.05, 1.13)	1.05 (1.02, 1.09)	1.07 (1.04, 1.09)	1.03 (1.01, 1.04)	0.95 (0.93, 0.96)
Liver disease						
	1999	0.93 (0.83, 1.05)	0.83 (0.74, 0.92)	0.95 (0.87, 1.03)	0.96 (0.91, 1.02)	1.06 (1.02, 1.09)
	2019	1.02 (0.91, 1.14)	1.04 (0.95, 1.13)	1.02 (0.95, 1.10)	1.03 (0.97, 1.08)	0.97 (0.94, 1.01)
Renal failure						
	1999	0.98 (0.90, 1.06)	0.95 (0.88, 1.02)	0.93 (0.88, 0.99)	1.04 (1.00, 1.09)	1.01 (0.98, 1.04)
	2019	1.20 (1.12, 1.28)	1.13 (1.07, 1.19)	1.10 (1.05, 1.15)	1.05 (1.02, 1.09)	0.89 (0.87, 0.92)
Natural death						
	1999	1.05 (0.99, 1.11)	1.18 (1.12, 1.23)	1.16 (1.11, 1.21)	1.06 (1.03, 1.10)	0.90 (0.88, 0.92)
	2019	1.03 (1.01, 1.06)	1.00 (0.98, 1.02)	1.02 (1.00, 1.04)	0.95 (0.93, 0.96)	1.02 (1.01, 1.03)
Injury						
	1999	0.99 (0.92, 1.06)	1.13 (1.07, 1.19)	1.09 (1.04, 1.15)	1.05 (1.02, 1.09)	0.94 (0.92, 0.96)
	2019	1.10 (1.04, 1.18)	1.08 (1.03, 1.13)	1.10 (1.06, 1.15)	1.02 (0.99, 1.06)	0.93 (0.91, 0.95)
Suicide						
	1999	1.20 (1.10, 1.31)	1.13 (1.04, 1.21)	1.06 (0.99, 1.14)	0.92 (0.88, 0.97)	0.98 (0.96, 1.01)
	2019	1.06 (0.92, 1.21)	1.00 (0.91, 1.10)	1.00 (0.93, 1.08)	0.99 (0.93, 1.04)	1.00 (0.97, 1.04)

* SMR, standardized mortality ratio; CI, confidence interval.
